# m7GHub V2.0: an updated database for decoding the N7-methylguanosine (m^7^G) epitranscriptome

**DOI:** 10.1093/nar/gkad789

**Published:** 2023-10-09

**Authors:** Xuan Wang, Yuxin Zhang, Kunqi Chen, Zhanmin Liang, Jiongming Ma, Rong Xia, João Pedro de Magalhães, Daniel J Rigden, Jia Meng, Bowen Song

**Affiliations:** Department of Public Health, School of Medicine & Holistic Integrative Medicine, Nanjing University of Chinese Medicine, Nanjing 210023, China; Department of Biological Sciences, Xi’an Jiaotong-Liverpool University, Suzhou, 215123, China; Department of Biological Sciences, Xi’an Jiaotong-Liverpool University, Suzhou, 215123, China; Institute of Systems, Molecular and Integrative Biology, University of Liverpool, L7 8TX, Liverpool, UK; Key Laboratory of Ministry of Education for Gastrointestinal Cancer, School of Basic Medical Sciences, Fujian Medical University, Fuzhou 350004, China; Department of Biological Sciences, Xi’an Jiaotong-Liverpool University, Suzhou, 215123, China; Department of Biological Sciences, Xi’an Jiaotong-Liverpool University, Suzhou, 215123, China; Institute of Systems, Molecular and Integrative Biology, University of Liverpool, L7 8TX, Liverpool, UK; Department of Financial and Actuarial Mathematics, Xi’an Jiaotong-Liverpool University, Suzhou, 215123, China; Institute of Inflammation and Ageing, University of Birmingham, B15 2WB, Birmingham, UK; Institute of Systems, Molecular and Integrative Biology, University of Liverpool, L7 8TX, Liverpool, UK; Department of Biological Sciences, Xi’an Jiaotong-Liverpool University, Suzhou, 215123, China; Institute of Systems, Molecular and Integrative Biology, University of Liverpool, L7 8TX, Liverpool, UK; AI University Research Centre, Xi’an Jiaotong-Liverpool University, Suzhou 215123, China; Department of Public Health, School of Medicine & Holistic Integrative Medicine, Nanjing University of Chinese Medicine, Nanjing 210023, China

## Abstract

With recent progress in mapping N7-methylguanosine (m^7^G) RNA methylation sites, tens of thousands of experimentally validated m^7^G sites have been discovered in various species, shedding light on the significant role of m^7^G modification in regulating numerous biological processes including disease pathogenesis. An integrated resource that enables the sharing, annotation and customized analysis of m^7^G data will greatly facilitate m^7^G studies under various physiological contexts. We previously developed the m7GHub database to host mRNA m^7^G sites identified in the human transcriptome. Here, we present m7GHub v.2.0, an updated resource for a comprehensive collection of m^7^G modifications in various types of RNA across multiple species: an m7GDB database containing 430 898 putative m^7^G sites identified in 23 species, collected from both widely applied next-generation sequencing (NGS) and the emerging Oxford Nanopore direct RNA sequencing (ONT) techniques; an m7GDiseaseDB hosting 156 206 m^7^G-associated variants (involving addition or removal of an m^7^G site), including 3238 disease-relevant m^7^G-SNPs that may function through epitranscriptome disturbance; and two enhanced analysis modules to perform interactive analyses on the collections of m^7^G sites (m7GFinder) and functional variants (m7GSNPer). We expect that m7Ghub v.2.0 should serve as a valuable centralized resource for studying m^7^G modification. It is freely accessible at: www.rnamd.org/m7GHub2.

## Graphical Abstract

**Graphical Abstract:**
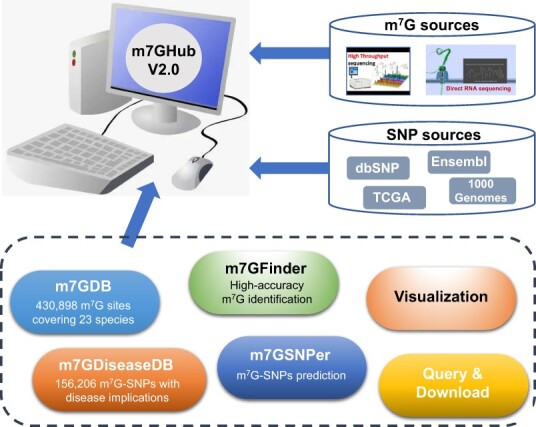

## Introduction

Over 170 types of chemical modification are naturally decorated on cellular RNAs of all three kingdoms of life, modulating various biological processes such as translation, RNA stability and RNA metabolism (1,2). Among them, N7-methylguanosine (m^7^G) is the most ubiquitous RNA cap modification added to the 5′ cap at the initial stage of transcription (3). Recent studies suggested that m^7^G capping modulates nearly the entire life cycle of messenger RNA (mRNA), including mRNA splicing (4), translation (5), RNA processing and metabolism (6) and transcription (7), and influences various cellular processes including gene expression and transcript stabilization (8). Additionally, the presence of m^7^G modification in ribosomal RNA (rRNA) (9) and transfer RNA (tRNA) (10) has also been reported, and mutations that impair tRNA m^7^G methylation found to cause microcephalic primordial dwarfism (11).

We previously developed an integrated resource m7GHub to share data on m^7^G RNA modification in the human transcriptome (12). In the first release, m7GHub collected 44 058 experimentally validated human mRNA m^7^G sites and 57 769 m^7^G-associated variants, respectively. Additionally, 1218 m^7^G disease-relevant m^7^G-SNPs were further annotated, with implications for the potential pathogenesis of ∼600 disease phenotypes.

To date, several high-throughput sequencing techniques have been developed and applied for transcriptome-wide profiling of m^7^G RNA modification. The m^7^G-MeRIP-seq was first introduced in 2019 to profile m^7^G distribution in human and mouse transcriptome, respectively (13). This antibody-based immunoprecipitation technique reveals m^7^G-containing regions with a resolution ∼100 bp and has since been further applied to multiple species including rat and zebra fish (14–16). By combining the conventional MeRIP-seq approach with ultraviolet cross-linking, m^7^G-miCLIP-seq achieved an improved resolution of ∼30 bp (17). In addition, base-resolution approaches such as m^7^G-seq (13) and m^7^G-MaP-seq (18) offer the precise location of m^7^G modification sites. Several overall patterns of m^7^G modification sites have also been reported across profiling techniques. Specifically, statistically significant GA- or GG-enriched motifs were identified in peaks using m7G-MeRIP-seq (13), while AG-rich contexts were reported from m7G-miCLIP-seq (17). Additionally, diverse sequence motifs around base-resolution m^7^G sites have also been reported by m7G-seq, with G(m^7^G)A and A(m^7^G)A ranking the top two motifs. Taken together, these findings suggested that additional methyltransferase(s) may be involved for m^7^G installation (13). Besides next-generation sequencing (NGS)-based methods, the newly emerged direct RNA sequencing platform developed by Oxford Nanopore Technology (ONT) also provides a promising alternative, allowing the simultaneous real-time identification of any natural modifications in the RNA molecule based on characteristic signals (19). Several pilot studies have offered specific or mixed identification of modified residues, such as m6Anet (m^6^A) (20), MINES (m^6^A) (21), nanoPsu (pseudourindine) (22), ELIGOS (mixed) (23) and Tombo (mixed). The ELIGOS and Tombo studies report a set of putative modified residues without differentiating the modification type, but these unknown types of candidate modification site can be further labeled using deep learning models.

In response to our rapidly expanding knowledge in RNA modification, bioinformatics databases have been developed to share, annotate and interpret the generated datasets. These bioinformatics efforts include: MODOMICS for querying RNA modification pathways (24); RMBase v.2.0 to collect of RNA modification sites (25); RMVar for unveiling RNA modification (RM)-associated variants (26); RM2Target for collection of writers, erasers and readers (WERs) of RNA modifications (27); m6A-Atlas as an m^6^A knowledgebase (28) and ConsRM for quantifying m^6^A conservation (29). However, to the best of our knowledge, resources for m^7^G-related knowledge are still limited to m7GHub.

**Figure 1.**
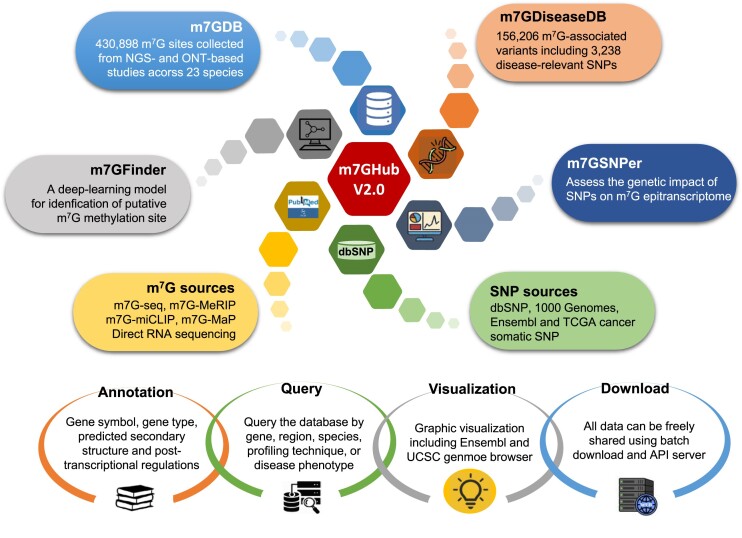
The overall construction of m7GHub v.2.0. The updated m7GHub v.2.0 consists of four major components: (i) m7GDB: the first m^7^G database containing ∼430 000 putative m^7^G sites collected from both NGS- and ONT-derived samples; (ii) m7GFinder: a deep learning-based high accuracy m^7^G predictor covering m^7^G identification in four different species; (iii) m7GSNPer: a real-time analysis module to assess the impact of genetic variants on database collection; (iv) m7GDiseaseDB: a database holding ∼150 000 functional variants involved in m^7^G modification, with implications for the potential pathogenesis of ∼1300 known phenotypes. An integrated web interface offers query, search, visualize and download function of all collected data is freely accessible at: www.rnamd.org/m7GHub2.

In this study, we have upgraded m7GHub to version 2.0 by integrating all recently identified m^7^G RNA modification sites derived from NGS and ONT-based studies, from which m^7^G-affecting variants were revealed using a deep learning model. The m7GHub v.2.0 consists of the following major updates: (i) m7GDB: a comprehensive m^7^G database consisting of 258 206 NGS-based m^7^G sites and the first collection of 172 692 putative m^7^G sites derived from ONT samples with rich functional annotations, covering a total of 23 species. (ii) m7GDiseaseDB: a database holding the most complete collection of 156 206 m^7^G-associated variants that may add or remove an m^7^G methylation site, with 3238 disease-relevant variants that may shed light on disease mechanisms acting through epitranscriptome layer circuitry. (iii) Enhanced modules allow interactive analysis of the database collections and user-uploaded datasets, from which putative m^7^G sites (m7GFinder) and epitranscriptome disturbance (m7GSNPer) of user-interested genome regions/genetic variants can be determined. The overall design of m7GHub v.2.0 is outlined in Figure 1. We expect that m7GHub v.2.0 will be a valuable one-stop platform for researchers who are interested in m^7^G modification: it is freely accessible at: www.rnamd.org/m7GHub2.

## Materials and methods

### Collection of m^7^G sites based on profiling techniques

The m^7^G sites collected in m7GHub v.2.0 were derived from both high-throughput sequencing (NGS) and Oxford Nanopore direct RNA sequencing (ONT) samples. Regarding NGS-based studies, the m^7^G sites were obtained from 74 sequencing samples using five different m^7^G profiling techniques. Additionally, 116 direct RNA sequencing samples, comprising 42 FAST5 and 74 FASTQ files, were collected from 37 independent studies in the NCBI GEO database (Supplementary Tables S1 and S2). Specifically, the collected m^7^G sites were classified into three different groups as illustrated next:

1. **NGS techniques (base-resolution)**: the m^7^G sites classified in this group were extracted from NGS-based studies at base-resolution level. The genome coordinates of m^7^G residues were extracted from the relating GSE or corresponding supplementary files of m7G-seq and m7G-MaP-seq studies, respectively. For m7G-seq, we re-processed the raw sequencing data to map the base-resolution m^7^G sites to human genome assembly hg38, following the same protocol implemented in the original study (13).
2. **NGS techniques (m^7^G-containing region)**: the m^7^G-containing regions were extracted from m7G-MeRIP-seq (∼150 bp) and m7G-miCLIP-seq (∼30 bp), respectively. Specifically, the m^7^G-containing regions from m7G-MeRIP-seq were obtained using a common pipeline. The raw FASTQ datasets were directly downloaded from NCBI Gene Expression Omnibus (GEO) (30), the raw reads were trimmed and aligned to the reference genome using HISAT2 (31), and peak-calling process was implemented by exomePeak2 (32). Besides m7G-MeRIP-seq, the genome coordinates of m^7^G-containing regions from m7G-miCLIP-seq were extracted from the supplementary files of its original study (17).
3. **ONT-derived and deep-learning prediction**: to try to unveil the landscape of m^7^G methylation generated by direct RNA sequencing techniques, we obtain the ONT-based m^7^G sites by large-scale prediction of modified guanosines using our previously developed deep neural network models (33). As no tools were available for specifically predicting m^7^G sites from direct RNA sequencing data, the Tombo and ELIGOS were used to screen out all non-canonical guanosines from direct RNA sequencing samples. Specifically, the raw FAST5 data were re-squiggled with the ‘Tombo re-squiggle’ module and candidate modification sites were detected by the ‘Tombo de novo modification detection’ module based on signal shifts. ELIGOS used the base calling errors (i.e. insertion, deletion, substitution and decreased base call qualities) caused by the presence of non-canonical bases. Raw FAST5 data were base called with Guppy and aligned to their reference genome with Minimap2. Then, ELIGOS extracted the base call error profile from the alignment SAM file and compared it with expected one. Sites with significantly higher errors were reported as potential modification sites. Consequently, Tombo and ELIGOS reported a set of putative modified guanosines without differentiating their modification type. The modified guanosines were further assessed by our previously developed neural network (33), trained on the NGS-validated m^7^G sites from four species (human, mouse, rat and zebra fish), respectively. Only the modified guanosines passing a strict cut-off (average prediction score >0.5 and upper bound of *P*-value < 0.05) were retained as putative m^7^G sites and included in the m7GDB database.

### Evaluating the epitranscriptome impact of genetic variants on m^7^G methylation status

In this study, two types of genetic variant were considered to assess their epitranscriptome impact on m^7^G methylation status. The germline variants were extracted from dbSNP (v151) (34), 1000 Genomes (Phase 3 Mitochondrial Chromosome Variants set) and Ensembl 2022 (Ensembl release 106) (35). In addition, 33 different cancer types of human somatic variants were collected from the Cancer Genome Atlas (TCGA) (release v.35) (36). Together, a total of 6 0826 918 germline variants and 2 264 915 somatic variants identified in four species were included, and the detailed datasets of genetic variants analyzed in this study can be found in Supplementary Table S3.

Following the well-defined definition for predicting m^7^G-affecting variants in m7GHub and other related studies (26,37), an m^7^G-associated variant was characterized based on its ability to cause the gain or loss of an m^7^G modification site, as predicted by our previously described deep neural network models (33). Three different confidence levels were further defined: (i) high: a genetic variant directly altered an experimentally validated m^7^G site at base-resolution level (m7G-seq or m7G-MaP-seq), leading to the loss of the modified nucleotide; (ii) medium: a genetic variant altered a nucleotide within the 41-nt flanking window of a base-resolution m^7^G site or within an m^7^G-containing region (∼30–150 nt, identified by m7G-MeRIP-seq or m7G-miCLIP-seq), resulting in the loss of an m^7^G status in the mutated sequence, as determined by the deep learning model and (iii) low: the low confidence level covers the transcriptome-wide prediction for reference- and mutated-sequence (altered by a genetic variant) around guanosines, the significant decrease or increase in the m^7^G probability were reported by the deep learning model to define m^7^G-loss or m^7^G-gain mutation, respectively. Specifically, we calculated the association level (AL) between genetic variant and m^7^G site as follows:


(1) \begin{equation*}{\mathrm{AL}} = \left\{ {\begin{array}{@{}*{2}{c}@{}} {{\mathrm{2}}{P}_{SNP} - 2\max \left( {0.5,{P}_{WT}} \right)}&{{\mathrm{for\, gain}}}\\ {2{P}_{WT} - 2\max \left( {0.5,{P}_{SNP}} \right)}&{{\mathrm{for\, loss}}} \end{array}} \right.\end{equation*}

Where the association level (AL) was calculated based on the probability of m^7^G methylation status for reference (wide type, ${P}_{WT}$) and mutated sequence (SNP altered, ${P}_{SNP}$) ranging from 0 to 1, with a value of 1 indicating the greatest epitranscriptome impact of the genetic variants on m^7^G status. The statistical significance was assessed by comparison to the ALs of all genetic variants, from which we use the upper bound of the *P*-value to represent the absolute ranking of each m^7^G-associated variant. Only the variants with a *P*-value < 0.05 (within the top 5% ALs of all genetic variants) were retained in the database collection.

### Functional annotation for m^7^G sites and m^7^G-associated variants

Functional annotations were integrated to help better interpretate the regulatory roles of the m^7^G epitranscriptome. The collected m^7^G sites and functional variants were first annotated with basic information such as gene annotation, transcript structure and predicted RNA secondary structure information (38). The potential involvement of post-transcriptional regulations was addressed with data collected from POSTAR2 (39) (RBP binding regions), miRanda (40) and startBase2 (41) (miRNA–RNA interaction), and UCSC browser (42) annotation (GT-AG splicing sites). In addition, the m^7^G-associated variants were annotated with mutation type (nonsynonymous or synonymous variant), TCGA barcode, RS ID, deleterious level (predicted by five independent scores (43–46)). This information was derived from the ANNOVAR package (47), dbSNP (34) and the TCGA database (36).

### Potential involvement of m^7^G methylation in disease pathogenesis

A large number of disease-related variants (TagSNPs) were obtained from ClinVar (48), the GWAS catalog (49) and Johnson and O’Donnel's database (50). In addition, the TagSNPs were used to implement linkage disequilibrium (LD) analysis using PLINK (51) tool (parameters: –r2 –ld-snp-list –ld-window-kb 1000 –ld-window 10 –ld-window-r2 0.8). The disease TagSNPs and their LD mutations were mapped to all m^7^G-associated variants to explore the potential pathogenesis of known disease-phenotypes through m^7^G regulation.

### Database and web interface implementation

Hyper text markup language (HTML), cascading style sheets (CSS) and hypertext preprocessor (PHP) were used in the fundamental development of m7GHub v.2.0 web interfaces. We implemented MySQL and ECharts to present metadata and statistical diagrams, respectively. Additionally, the interactive exploration of user-interested genome coordinates were visualized by JBrowse genome browser (52).

## Results

### m^7^G sites collected in m7GDB

The updated m7GDB database holds a total of 430 898 m^7^G sites (see Table 1) collected from NGS- and ONT-based studies, representing a significant expansion in both number of collected m^7^G sites (∼10-fold expansion) and covered species (from human only to 23 species) compared to the first release. Specifically, the NGS-derived m^7^G sites cover seven species including human (169 718), mouse (18 595), rat (49 440), zebra fish (20 342), yeast (88), *Arabidopsis* (19) and *Escherichia coli* (4). For the human collection, the m^7^G sites were further classified according to their profiling techniques, including base-resolution level (8402 sites, m7G-seq and m7G-MaP-seq) and m^7^G-containing region (22 783 sites, m7G-miCLIP-seq, ∼30 bp; 138 534 sites, m7G-MeRIP-seq, ∼150 bp). For datasets collected from direct RNA sequencing studies, a total of 172 692 modified guanosines annotated with m^7^G probability were collected across 21 species at base-resolution level, such as human (76 077), mouse (13 828), fruit fly (298), pig (366), maize (8939) and *Arabidopsis* (3083). In particular, the m^7^G epitranscriptome in 20 species is covered for the first time, and data from direct RNA sequencing samples included. Compared to the previous version and other epitranscriptomic databases (RMBase (25), RMVar (26) and RMDisease (37)), m7GHub represents the most comprehensive knowledgebase for collections of m^7^G methylation so far (Table 2).

**Table 1.** Collection of m^7^G sites in m7GDB

	Experimentally validated NGS techniques		ONT-derived and deep-learning prediction	
Species	1bp	∼30–300 bp	1bp	Total
Human	8402	161 316	76 077	245 795
Mouse	/	18 595	13 828	32 423
Rat	/	49 440	/	49 440
Zebra fish	/	20 342	/	20 342
19 other species	111	/	82 787	82 898
*Total*	8513	249 693	172 692	430 898

**Table 2.** Comparison of m7GHub v2.0 with other epitranscriptome databases

	m7GHub v.2.0	m7GHub v1.0 (12)	RMDisease v.2.0 (37)	RMVar (26)	RMBase v2.0 (25)	DirectRM DB (53)
Number of m^7^G sites collected	430 898	44 058	9365	43 367	318	1189
Covered species (m^7^G site)	23	1	1	2	3	1
Number of m^7^G-associated SNP	156 206	57 769	24 049	64 867	7	/
Covered species (m^7^G-SNP)	4	1	1	2	1	/
Disease-associated m^7^G-SNP	3238	1218	507	861	/	/
Interactive analyses (m^7^G site identification)	Yes (4 species)	1	/	/	/	/
Interactive analyses (m^7^G-SNP identification)	Yes (4 species)	1	1	/	/	/

### Potential disease pathogenesis involving m^7^G disturbance (m7GDiseaseDB)

m7GDiseaseDB holds a total of 156 206 genetic variants that may add or remove m^7^G methylation status in four species (Table 3), including human (97 407), mouse (23 564), rat (7422) and zebra fish (27 813), providing the most comprehensive map of genetic factors potentially relating to m^7^G disturbance so far. To unveil the potential mechanisms of disease phenotypes functioning at the epitranscriptome layer, we then mapped all collected human m^7^G-associated variants to pathogenic TagSNPs and their LD mutations. We found that 3238 m^7^G-associated variants localized on 1651 genes were recorded with 1308 known disease phenotypes, which is nearly three times the number in the previous version. Additionally, 64 266 m^7^G-associated variants were also derived from TCGA cancer somatic mutations, revealing the potential involvement of m^7^G methylation in 33 types of human cancer. Finally, we identified the disease phenotypes and TCGA cancer types that are most strongly linked with m^7^G disturbance (Supplementary Table S4).

**Table 3.** m^7^G-associated variants collected in m7GDiseaseDB

		m^7^G-associated variants			ClinVar			GWAS		
Species	Confidence level	Loss	Gain	Total	SNP	Disease	Gene	SNP	Disease	Gene
Human (Germline SNP)	High	1316	/	1316	94	92	90	24	22	24
	Medium	9699	/	9699	660	361	413	107	73	97
	Low	7518	14 608	22 126	840	515	581	256	141	240
Human (Somatic SNP)	High	4018	/	4018	92	113	83	5	5	5
	Medium	30 073	/	30 073	569	309	322	53	41	48
	Low	7911	22 264	30 175	538	349	393	53	41	51
Mouse	High	530	/	530	/	/	/	/	/	/
	Medium	4055	/	4055	/	/	/	/	/	/
	Low	7595	11 384	18 979	/	/	/	/	/	/
Rat	Medium	4225	/	4225	/	/	/	/	/	/
	Low	1694	1503	3197	/	/	/	/	/	/
Zebra fish	Medium	7285	/	7285	/	/	/	/	/	/
	Low	10 647	9881	20 528	/	/	/	/	/	/

**Note:** The TCGA somatic variants were extracted from 33 different types of human cancer projects. The m^7^G-associated variants classified into high confidence level refer to mutations directly destroying base-resolution modified nucleotides (m^7^G site). The numbers in the ‘ClinVar’ and ‘GWAS’ sections represent the number of m^7^G-associated variants mapped to the disease-related TagSNPs having ClinVar or GWAS records, respectively.

### Enhanced web interface and usage

The web interface of m7GHub v.2.0 has been re-designed to present an informative, fast and user-friendly one-stop knowledgebase for m^7^G study, which enables users to quickly query, carry out customized searches of and freely download all collected datasets. Four major modules were presented in m7GHub, namely m7GDB, m7GDiseaseDB, m7GFinder and m7GSNPer.

#### m7GDB

**Figure 2.**
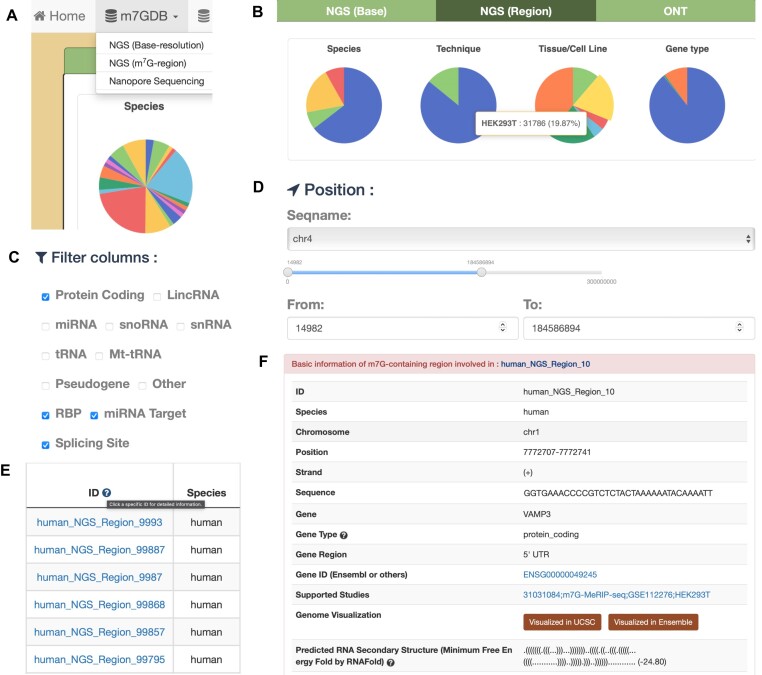
Contents of m7GDB. (**A** and **B**) The m^7^G sites collected in m7GDB were classified into three different group according to their profiling techniques; users can briefly check the statistical distribution of collected data summarized by pie charts. (**C** and **D**) Several options were provided to further filter the datasets, including a position par to extract specific genomic region of interests. (**E** and **F**) Once customized filtering has been applied, the user can click the site ID to view the detailed information of a specific m^7^G site.

The experimentally validated m^7^G sites were collected in m7GDB module. Users can visualize the landscape of m^7^G modification in different species according to the profiling techniques (Figure 2A). For example, users can query the deposited m^7^G-containing region by clicking the ‘NGS (m^7^G region)’ button on the top menu bar, the returned page first summarizes the statistical distribution of collected m^7^G region categorized by species, profiling technique, tissue/cell line and gene type (Figure 2B). Various filters allow users to further filter their data of interest, including gene type and involvement of post-transcriptional regulation (Figure 2C). In addition, a position bar offers a function to extract customized regions of user interest (Figure 2D). The returned results exclusively display m^7^G sites that satisfy all selected filter options (Figure 2E): users can simply click on the site ID to access detailed information about a specific m^7^G site (Figure 2F).

#### m7GDiseaseDB

**Figure 3.**
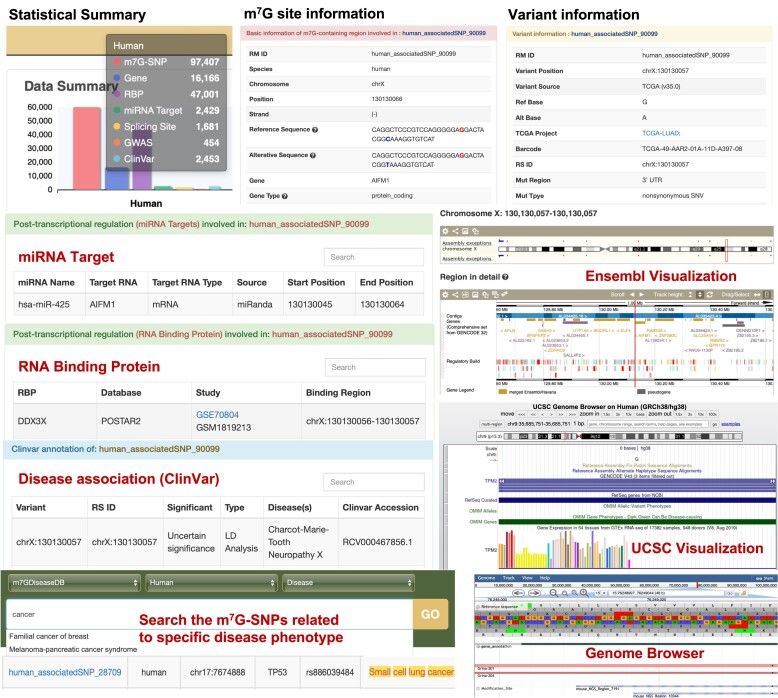
Enhanced web interface of m7GDiseaseDB. User can query the collected m^7^G-SNPs by selecting a species and check the summary table. Users can further click the individual RM ID to access the basic information of the associated m^7^G-SNP and involved m^7^G site. The web-interface also features various graphic visualizations including Ensembl and UCSC genome browser, especially useful for presentation of SNP information. In addition, the disease associations involved m^7^G methylation can be extracted by searching a specific disease or phenotype.

The m^7^G-associated variants and disease associations were collected in m7GDiseaseDB (Figure 3), from which users can query each m^7^G-associated SNP with detailed annotations such as reference sequence, mutated sequence, relative position of SNP, potential involvement in post-transcriptional regulation (miRNA targets, RBP binding, splicing events), crosslinks to dbSNP/GtRNAdb and their epitranscriptome effects on m^7^G status (gain or loss function). The disease associations can be obtained by clicking ‘GWAS’ or ‘ClinVar’ buttons from the filter columns. In addition, the ‘Disease’ option on the search box allows users to query all m^7^G-associated variants linking to a specific disease phenotype, along with other search options such as gene symbol, genome coordinate and RS ID. Finally, the m7GDiseaseDB also offers various graphic visualizations that displaying the position of the m7G-SNPs along the gene and genomic regions of interest, such as Ensembl and UCSC genome browser.

#### Analysis modules (m7GFinder and m7GSNPer)

**Figure 4.**
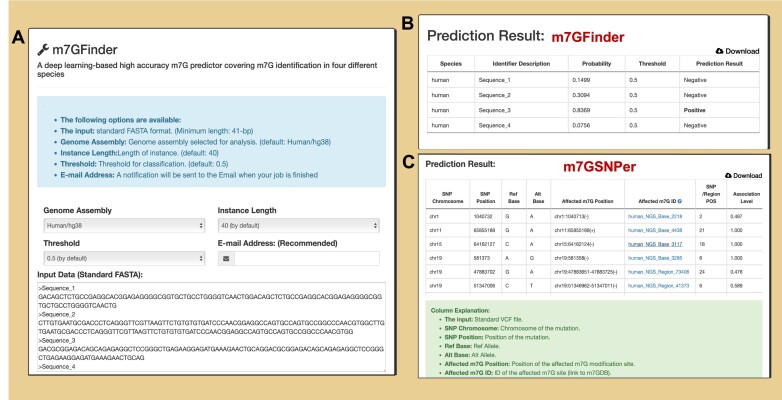
Contents of m7GFinder and m7GSNPer. **(A)** Web interface of m7GFinder. **(B)** Prediction results from m7GFinder. The m7GFinder reports the prediction label at the bag level (the entire input sequence), rather than a specific nucleotide. **(C)** Prediction results from m7GSNPer. The explanation for each column has been presented clearly, and the data is available for free download and sharing.

To allow users to perform interactive analyses on the collected datasets, two enhanced modules are presented based on our previously developed deep neural network models (33). The m7GFinder was developed for high-accuracy prediction of putative m^7^G sites from user-uploaded RNA sequences (standard FASTA format). A minimum sequence length of 41 nt is required as input data (Figure 4A). The multi-instance learning framework treats each entire input sequence as a ‘bag’ and reports its bag-level label (m^7^G probability). Importantly, the m7GFinder reports the prediction label at the bag level (the entire input sequence), rather than a specific nucleotide (Figure 4B). Consequently, each input sequence with a length around 150 nt (typical length of most m^7^G peaks from MeRIP-seq) is recommended. Besides m7GFinder, the m7GSNPer module allows users to evaluate the associations between SNPs of their interest and the m^7^G epitranscriptome of a specific species. The standard VCF file containing a group of genetic variants is acceptable as input data for m7GSNPer, with the association level (AL) was calculated between reference and mutated sequences. The returned results of m7GSNPer can be freely downloaded with detailed column explanations (Figure 4C).

#### Batch download and API server

Two downloading options are provided for all datasets collected in m7GHub v.2.0. (i) Multiple datasets can be simultaneously selected for batch downloading on the ‘Download’ page. (ii) The application program interface (API) server provides a highly flexible download option: instructions and examples to access the API server are provided on ‘API’ page.

## Discussion

With the rapid accumulation of sequencing samples derived from NGS and ONT technologies, comprehensive maps of m^7^G modifications under various biological contexts have been revealed. We have updated m7GHub to version 2.0, an all-in-one online platform designed to effectively store, annotate, analyze and share the m^7^G data. Compared to the first release (m7GHub v1.0) and other epitranscriptome databases, our updated version covered so far the most comprehensive collections of m^7^G-related data (please refer to Table 2), including: (i) a comprehensive database (m7GDB) of 430 898 previously reported m^7^G sites, including the first collection of putative m^7^G sites from ONT-derived samples, marking an 10-fold expansion compared with the first release. (ii) m^7^G-associated variants identified in four species, of which potential involvement in 1308 disease phenotypes was revealed for 3238 disease-related m^7^G-affecting SNPs (m7GDiseaseDB). In addition, two deep learning-based analysis tools (m7GFinder and m7GSNPer) were developed to support analyses of the database or user-uploaded data.

In conclusion, m7GHub v.2.0 offers an extensive repository of m^7^G epitranscriptome data across various species. However, in the current version, the landscape of putative m^7^G modification from direct RNA sequencing samples was predicted by deep-learning model of modified guanosines, and thus only offers limited reliability. With the rapid advancement and widespread adoption of direct RNA sequencing techniques, we can expect the development of software to directly identify m^7^G modifications from direct RNA sequencing samples in the near future. Additionally, due to variations in the number of sequencing samples across different species, the m^7^G sites currently collected in the database cannot directly represent the overall distribution of m^7^G modification in a given species, especially for species with extremely limited sequencing samples available (e.g. yeast and *E. coli*). Consequently, the database will undergo regular updates by continuously incorporating the latest sequencing data and methodologies to ensure it remains a useful resource for the m^7^G research community.

## Supplementary Material

gkad789_Supplemental_Files

## Data Availability

The raw data used to develop m7GHub v.2.0 is already publicly available in the NCBI GEO database, The Cancer Genome Atlas (TCGA release v.35), dbSNP (v.151), 1000 Genome and Ensembl 2022 (Ensembl release 106). The detailed description (accession number) can be found in Supplementary Tables S1–S3. All data collected in m7GHub v.2.0 is freely accessible at: www.rnamd.org/m7GHub2.
