# Supplementary material for: m7GHub V2.0: an updated database for decoding the N7-methylguanosine (m7G) epitranscriptome
Source: Nucleic Acids Res. 2023 Oct 9;52(D1):D203–12. doi: 10.1093/nar/gkad789 (PMC10767970; doi:10.1093/nar/gkad789)
Supplement: gkad789_Supplemental_Files [file gkad789_supplemental_files.zip › Supplementary information.docx]

**Table S4. The disease phenotypes and TCGA cancer projects that**

**most enriched with m^7^G-SNP**

| ClinVar & GWAS | | | | | |  | TCGA | |
| --- | --- | --- | --- | --- | --- | --- | --- | --- |
| Name | # | Database | Study  accession | Clinical significance | Identifier |  | Project name | Ratio  (m7G-SNPs  /Total SNP) |
| Hereditary cancer-predisposing syndrome | 61 (1.88%) | ClinVar | RCV000164762.2 | Pathogenic | MedGen:  C0027672 |  | Cervical Squamous Cell Carcinoma and Endocervical Adenocarcinoma  (TCGA-CESC) | 3.95% |
| Primary ciliary dyskinesia | 28  (0.86%) | ClinVar | RCV000352096.1 | Likely Benign | MedGen:  C0008780 |  | Pheochromocytoma and Paraganglioma (TCGA-PCPG) | 3.83% |
| Cardiovascular phenotype | 27  (0.83%) | ClinVar | RCV000247933.1 | Benign | MedGen:  CN230736 |  | Mesothelioma  (TCGA-MESO) | 3.82% |
